# *Toxoplasma gondii* Effects on the Relationship of Kynurenine Pathway Metabolites to Acoustic Startle Latency in Schizophrenia vs. Control Subjects

**DOI:** 10.3389/fpsyt.2020.552743

**Published:** 2020-11-20

**Authors:** Bradley D. Pearce, Nicholas Massa, David R. Goldsmith, Zeal H. Gandhi, Allison Hankus, Alaaeddin Alrohaibani, Neha Goel, Bruce Cuthbert, Molly Fargotstein, Dana Boyd Barr, Parinya Panuwet, Victoria M. Brown, Erica Duncan

**Affiliations:** ^1^Rollins School of Public Health, Emory University, Atlanta, GA, United States; ^2^Atlanta Veterans Affairs Health Care System, Decatur, GA, United States; ^3^Department of Psychiatry and Behavioral Sciences, Emory University School of Medicine, Atlanta, GA, United States

**Keywords:** schizophrenia, acoustic startle, *Toxoplasma gondii*, kynurenines, startle latency, tryptophan

## Abstract

**Background:** Chronic infection with *Toxoplasma gondii* (TOXO) results in microcysts in the brain that are controlled by inflammatory activation and subsequent changes in the kynurenine pathway. TOXO seropositivity is associated with a heightened risk of schizophrenia (SCZ) and with cognitive impairments. Latency of the acoustic startle response, a putative index of neural processing speed, is slower in SCZ. SCZ subjects who are TOXO seropositive have slower latency than SCZ subjects who are TOXO seronegative. We assessed the relationship between kynurenine pathway metabolites and startle latency as a potential route by which chronic TOXO infection can lead to cognitive slowing in SCZ.

**Methods:** Fourty-seven SCZ subjects and 30 controls (CON) were tested on a standard acoustic startle paradigm. Kynurenine pathway metabolites were measured using liquid chromatography-tandem mass spectrometry were kynurenine (KYN), tryptophan (TRYP), 3-hydroxyanthranilic acid (3-OHAA), anthranilic acid (AA), and kynurenic acid (KYNA). TOXO status was determined by IgG ELISA.

**Results:** In univariate ANCOVAs on onset and peak latency with age and log transformed startle magnitude as covariates, both onset latency [F_(1,61)_ = 5.76; *p* = 0.019] and peak latency [F_(1,61)_ = 4.34; *p* = 0.041] were slower in SCZ than CON subjects. In stepwise backward linear regressions after stratification by Diagnosis, slower onset latency in SCZ subjects was predicted by higher TRYP (B = 0.42; *p* = 0.008) and 3-OHAA:AA (B = 3.68; *p* = 0.007), and lower KYN:TRYP (B = −185.42; *p* = 0.034). In regressions with peak latency as the dependent variable, slower peak latency was predicted by higher TRYP (B = 0.47; *p* = 0.013) and 3-OHAA:AA ratio (B = 4.35; *p* = 0.010), and by lower KYNA (*B* = −6.67; *p* = 0.036). In CON subjects neither onset nor peak latency was predicted by any KYN metabolites. In regressions stratified by TOXO status, in TOXO positive subjects, slower peak latency was predicted by lower concentrations of KYN (B = −8.08; *p* = 0.008), KYNA (B = −10.64; *p* = 0.003), and lower KYN:TRYP ratios (B = −347.01; *p* = 0.03). In TOXO negative subjects neither onset nor peak latency was predicted by any KYN metabolites.

**Conclusions:** KYN pathway markers predict slowing of startle latency in SCZ subjects and in those with chronic TOXO infection, but this is not seen in CON subjects nor TOXO seronegative subjects. These findings coupled with prior work indicating a relationship of slower latency with SCZ and TOXO infection suggest that alterations in KYN pathway markers may be a mechanism by which neural processing speed, as indexed by startle latency, is affected in these subjects.

## Introduction

Schizophrenia (SCZ) is a devastating disease that often confers lifelong symptoms and disability upon its victims. Despite many decades of intensive research, the etiology and pathophysiology of this disorder remain perplexing. It is generally accepted that the etiology involves a complex relationship between genetic and environmental factors. Amongst environmental factors, interest in infectious illnesses as potential contributors to SCZ pathophysiology is gaining momentum (1, 2).

*Toxoplasma gondii* (TOXO) is a protozoan parasite that is able to infect most warm-blooded animals. It has a worldwide distribution and is the most common protozoan infection in the developed world (3). In the United States, ~13–20% of the population has been infected with TOXO (4, 5). Cats and other felines are the definitive hosts, but humans are among other mammals that serve as intermediate hosts (6, 7). Once a host is infected, the TOXO organism resides as microcysts in muscle and brain and likely remains there for the life of the host (6, 8, 9). Chronic TOXO infection is typically asymptomatic in immune competent people. However, importantly, two meta-analyses have shown that the risk for TOXO seropositivity was higher in patients with SCZ than in the general population with an odds ratio of 2.7; this finding was highly significant (95% confidence interval, 2.10–3.60; *p* < 0.000001) (10, 11). Since the original meta-analysis, the TOXO-SCZ association was replicated with 15 additional studies (12). Furthermore, otherwise asymptomatic TOXO infection is associated with cognitive impairments in human subjects, although the relationship is complex and may involve only certain cognitive domains (13–18).

There is a general consensus that an important mechanism by which an animal or human keeps chronic TOXO in check involves the tryptophan-kynurenine (KYN) pathway. TOXO infection elicits an induction of the cytokine interferon gamma (IFNγ), which in turn inhibits TOXO replication by depletion of tryptophan (TRYP) (7). The TOXO organism needs TRYP from the host milieu since it cannot synthesize this amino acid itself, and interferon gamma achieves local TRYP depletion by facilitating its degradation along the KYN pathway. Specific levels of downstream KYN metabolites depend in part on the location and activity of the enzymes along the pathway (19). KYN can be converted directly into kynurenic acid (KYNA), or down an alternative pathway yielding anthranilic acid (AA), 3-hydroxyanthranilic acid (3-OHAA) and finally into quinolinic acid (QUIN).

The acoustic startle response is a well-researched reflex following a sudden loud sound. It has been extensively researched in humans and in animal models of psychiatric disease, in part because virtually the same paradigm can be administered to rodents, monkeys, and humans. Preclinical research indicates that it is mediated by a simple subcortical neural circuit (20, 21). Latency of startle is the time between a startle eliciting stimulus and the response and is measured easily with millisecond (ms) precision. Because it is a pre-attentive reflexive response mediated by this simple circuit, it has been proposed as a putative measure of general speed of neural transmission (22). Latency is significantly slower in SCZ than control (CONT) subjects in most studies that have examined it (22–30), although some of the above studies only detected slowing of latency in trials with prepulse stimuli (23, 25, 29). There are a small number of studies that found no difference between SCZ and CON subjects (24, 31–33). Slower latency at baseline predicted which adolescent and young adult subjects at high risk for developing SCZ went on to convert to psychosis over a 2-years period in a multisite study of prodromal SCZ (34). Importantly for the current paper, latency was slower in TOXO seropositive than seronegative subjects, and this finding was evident both in SCZ and CON subjects in the cohort (35).

In further support of the relevance of startle latency to SCZ pathophysiology is emerging evidence that slower latency associates with poorer cognitive performance (26). We speculate that slowing of neural processing speed as indicated by slowing of startle latency could result in dysfunction of neural circuits that subserve cognition. Poorer cognitive performance, in turn, was associated with perturbations in molecules of the KYN pathway in humans (36). This is against a background of prior rodent work showing that stimulation of cortical kynurenic acid led to cognitive and sensory processing impairments (37–40), for which reason the KYN pathway has been proposed as a potential treatment target for cognitive deficits in SCZ (41). The current work was undertaken to investigate whether prolonged startle latency could be a mechanism by which KYN pathway alterations in the face of TOXO infection could lead to impaired cognition. To this end we assessed the relationship between kynurenine pathway metabolites and startle latency as a potential route by which chronic TOXO infection can lead to cognitive slowing in SCZ.

## Methods

### Subjects

Forty-seven SCZ subjects and thirty controls (CON) were enrolled in this cohort from the Atlanta Veterans Administration Health Care System and the surrounding community. To be included in this analysis the subject must have completed all assessments. Subjects in the SCZ group had a diagnosis of schizophrenia or schizoaffective disorder as verified by a structured diagnostic interview with the Structured Clinical Interview for DSM-IV, Axis-I (SCID) (42). SCZ subjects were excluded if they were currently being treated with clozapine. Both SCZ and CON subjects were excluded if they had a heart attack or heart failure in the previous 6 months, infection requiring antibiotics in the prior 60 days, hospitalization for any medical (non-psychiatric) condition in the prior 60 days, or any condition requiring systemic steroid treatment in the prior 60 days. Further, they were excluded if they had a medical diagnosis known to impact the immune system (such as HIV, AIDS, rheumatoid arthritis, etc.). Significant cognitive deficiencies or any history of neurological disease including head trauma with a loss of consciousness >5 min, overt CNS infection, neurovascular trauma, or seizure disorder were also exclusionary. Prior to entry into the study, subjects were screened for both vision and hearing to ensure that they had at least 20/30 acuity binocularly, and could hear at least 35 dB in all frequencies >500 Hz. Further, given a significant differential in rates of TOXO infection by country of birth (4), this study only included individuals born within the USA.

Participants were excluded if they tested positive for an illegal substance by urine toxicology performed on the day of enrollment. Cigarette smoking (and nicotine vaping) was allowed, although the amount of nicotine consumption was assessed by means of the Fagerstrom Smoking Tolerance Questionnaire (43).

### Startle Testing

Subjects participated in an acoustic startle session lasting 20 min where baseline startle magnitude and latency were evaluated using methods described in our previous work (22, 44), based on the standard startle protocol developed by Braff et al. (31). The eye-blink component of the acoustic startle reflex was measured *via* electromyography (EMG) of the right orbicularis oculi muscle. The three electrodes were placed as follows: 1 cm directly below the pupil of the right eye, 1 cm below the lateral canthus of the right eye, and on the mastoid behind the right ear. All resistances were <6 kΩ. EMG activity was recorded at 1-ms intervals for 250 ms following the onset of the startling stimulus for each trial. Subjects were seated in a sound attenuating audiology booth with eyes open and asked to look straight ahead for the duration of the test session.

Acoustic stimuli were delivered binaurally through headphones (Maico model TDH-39-P; Maico Diagnostics, Eden Prairie, MN). The startle paradigm began with 60 s of 70 dB white noise as an acclamation that continued as background for the remainder of the session. The startling stimuli were 116 dB white noise bursts, 40 ms in duration. Prepulse stimuli were 85 dB intensity white noise bursts of 20-ms duration. The session contained four trial types: pulse-alone trials, and prepulse+pulse trials with interstimulus intervals of 30, 60, or 120 ms between the prepulse and pulse stimuli. The session consisted of a habituation block of 6 pulse-alone stimuli, three blocks of 12 experimental stimuli, and concluded with another habituation block. Each experimental block consisted of three separate trials of each of the four trial types presented in pseudorandom fashion. Inter-trial intervals (ITI) were 10–25 s long with a mean of 15 s.

Data processing and reduction was carried out according to the methods of our prior work (22, 44). The signal was amplified and digitized using the computerized startle response monitoring system SR-Lab (San Diego Instruments, San Diego, CA). The signal was then full-wave rectified and subjected to a smoothing routine by the SR-Lab software that calculated a rolling average of 10 data points. The system calculated the baseline value as the average of the minimum and maximum EMG values recorded in the first 20 ms immediately following the startling stimulus. The onset of the blink response was defined as an increase of at least 7.33 μV (6 machine units) from the EMG value during the baseline period. Valid blink responses had to have an onset between 21 and 120 ms after the startling stimulus. To count as a valid blink, the peak magnitude of the blink response had to be a minimum of 12.21 μV (10 machine units) and had to occur no more than 95 ms after the onset of the response, and no more than 150 ms after the stimulus. The response magnitude was recorded as zero on trials in which the blink response was insufficient for scoring. For these trials, peak latency was also considered missing. Trials were discarded if the baseline EMG during the first 20 ms of recording was > 36.6 μV (30 machine units). For this paper we report analyses of onset and peak latency of the pulse-alone trials.

### Sample Collection, Metabolite, and TOXO Analysis

Human blood was collected into both serum tubes and in chilled EDTA-coated tubes. Upon collection of the sample all tube types were inverted 5-7 times. Serum tubes were allowed to clot for at least 30 min prior to centrifugation. Serum samples were centrifuged at 1,250 rcf for 10 min or until separation was achieved. EDTA samples remained on ice until they were centrifuged at 2,000 rcf for 15 min at 4°C. Both serum and plasma samples were aliquoted into 1 mL cryovials and stored at −80°C prior to analysis for TOXO and metabolites.

Serum specimens were analyzed for TOXO IgG antibodies as per manufacturer's instructions (Bio-Rad, Catalog# 25175, Redmond, WA). For all subjects, a discrete titer and dichotomous seropositivity status was determined using a three-point curve of the blank, a weakly positive calibrator, and a strongly positive calibrator. TOXO serointensity was determined from a direct calculation of absorbance against this three-point curve. A concentration of >33 IU/mL was indicative of TOXO seropositivity, whereas a value >27 IU/mL, but <33 IU/mL was indicative of equivocality. A concentration <27 IU/mL was an indicator of negative seropositivity. TOXO seropositivity status was classified dichotomously with 1 = TOXO seropositive and 0 = TOXO seronegative. Those who were positive and those who were equivocal were grouped together due to the fact that those with the oldest infections tend to have the lowest concentration of antibodies (45).

TRYP and its catalytic products KYN, KYNA, AA, and 3-OHAA were analyzed in plasma samples using liquid chromatography-tandem mass spectrometry (LC-MS/MS). Isotope dilution was also employed to increase the selectivity and sensitivity of the method. Prior to analysis, 200 μL of each plasma sample was spiked with an internal standard solution consisting of stable isotopically-labeled analogs of the target chemicals then the sample was mixed with 1 mL of 10% formic acid in water (v/v). The sample was loaded onto a Strata C18 solid phase extraction cartridge (500 mg/6 mL) (Phenomenex, Torrance, CA, USA) that had previously been conditioned with methanol and Milli-Q water. The cartridge was then washed with 1 mL of a mixture of methanol and Milli-Q water (10:90, v/v). The target compounds were eluted with 2 mL of methanol. The extract was evaporated to dryness under a gentle nitrogen stream and reconstituted with 100 μL of a mixture of methanol and water (1:49, v/v). A 2 μL volume of extract was injected onto an LC-MS/MS (Agilent Technology, Inc., Santa Clara, CA, USA). The target compounds were chromatographically separated using a Luna Pentafluorophenyl analytical column (3 μM, 4.6 × 100 mm) (Phenomenex). The mobile phase was Milli-Q water and methanol, both mixed with 1% formic acid. The target compounds were analyzed by a triple quadrupole MS (Agilent Technology, Inc.) using multiple reaction monitoring. The MS was operated in positive electrospray ionization mode. Nitrogen gas was used as a collision gas.

For each compound, two precursorproduct ion transitions were monitored, one of which was used for quantification and the other for confirmation. The quantification ions (m/z) were: 205188 (TRYP), 209192 (KYN), 154136 (3-OHAA), 190144 (KYNA), and 138120 (AA). One transition ion was monitored for each isotopically labeled analog that served as a reference standard and was used to automatically correct for extraction recovery.

Plasma samples were quantified using a 7-point, matrix-matched calibration curve ranging from 0.00391 to 3.906 μM. In each analytical run, the samples were analyzed alongside an analytical blank sample, as well as a low- and high-level quality control materials to ensure the proper operation of the analytical method. The method was successfully validated and had acceptable accuracy (i.e., within ±20%) and precision (i.e., <15% relative standard deviation) for all target compounds. The limits of detection were 3.906 μM for TRYP, 0.391 μM for KYN, and 0.00391 μM for KYNA, 3-OHAA, and AA. This method is able to generate values of the target compounds that are comparable to the ranges normally found in the human population.

### Statistical Analyses

Startle onset and peak latency and magnitude means and standard deviations were calculated for Block 1. We focused on Block 1 because in subsequent blocks some subjects had diminishing numbers of startle blinks due to habituation. Prior to statistical analysis all variables were analyzed for normality and corrected through log transformation when appropriate. The log transformed variables included TOXO IgG concentration, AA, KYNA, and startle magnitude. Race was coded as dichotomous variable by including those of “Other” race (multiracial) into the White population for our analyses (0 = White/Other, 1 = Black). TOXO status was coded as a dichotomous variable (0 = seronegative, 1 = seropositive). Diagnosis was coded as a dichotomous variable (0 = CON, 1 = SCZ). For demographic variables, Fisher's exact or Chi square tests for dichotomous variables and student's *t*-tests for continuous variables were used to determine any significant differences between CON and SCZ subjects. The difference in the rate of TOXO status (seropositive vs. seronegative) between the two subject groups was tested by means of Chi square analysis. For ANOVA and regression models described below, a *p* < 0.1 was required for any given predictor to be retained in the final models. To compare TOXO serointensity between CON and SCZ subjects, two separate univariate ANOVAs (one run on seropositives, one run on seronegatives) were used with age, race, and sex as additional predictors (only age and Diagnosis were retained in the final model).

As descriptive analyses, univariate ANOVAs were computed for KYN pathway variables, to compare CON and SCZ subjects. These models were initially run with age, race, sex, and TOXO status as predictors in addition to Diagnosis (CON vs. SCZ). The non-significant predictor, race, was omitted from the final models.

As additional descriptive analyses, similar univariate ANOVAs were used to compare startle variables between the CON and SCZ groups. For latency, initial models included age, race, sex, TOXO status (seropositive vs. seronegative) and startle magnitude. However, due to a lack of significance of race, sex, and TOXO status, only age and startle magnitude were retained in the final models. In the model for startle magnitude we initially included age, race, sex, TOXO status, but only Diagnosis (SCZ or CON) was retained in the final model due to non-significance of other covariates. We also ran these models to examine differences in startle variables between CON and SCZ with TOXO serointensity substituted for TOXO status as a covariate. The final models for latency retained age and startle magnitude in addition to Diagnosis as the only significant additional covariates. In the final model comparing startle magnitude between CON and SCZ subjects with TOXO serointensity, age, race and sex were not significant so were omitted.

Next, as tests of our primary hypothesis, we conducted backward stepwise linear regressions with KYN pathway variables as dependent measures and TOXO serointensity as a predictor variable. These models included Diagnosis (CON or SCZ) along with age, race, and sex as additional predictors. The final models omitted sex as a predictor due to non-significance.

As secondary tests of our hypotheses, stepwise backward linear regressions were completed on the cohort split by either Diagnosis (SCZ or CON) or by TOXO status (seropositive or seronegative). Onset and peak latency were the dependent variables in our analyses. Our independent variables were age, race, sex, startle magnitude, and each kynurenine metabolite individually. For models split by Diagnosis, TOXO was included as an independent variable both dichotomously (positive vs. negative), and continuously as a log serointensity. For models split by TOXO status, Diagnosis was included as an independent covariate in the model. All metabolites and their respective ratios were run in each model individually to avoid collinearity. All statistics were completed using either SPSS v25 (Armonk, NY) or SAS Studio (Cary, NC).

## Results

### Demographics and Ratings

Forty-seven SCZ subjects and 30 control subjects (CON) completed testing and are included in the cohort. Table 1 displays demographic and clinical variables for the sample. There was a significant difference between SCZ and CON subjects in race distribution (Fisher's exact test, *p* = 0.05). Age, sex distribution, and smoking status (smoker vs. non-smoker) did not differ significantly between CON and SCZ subjects.

**Table 1:** Demographic and clinical information by diagnostic group.

	**Control**	**Schizophrenia**	**Chi Sq/T**	***p*-Value**
			**value**	
*Demographics, n*	30	47		
**Sex**, ***n*** **(%)^a^**				
Male	25 (83.33)	42 (89.36)		0.500
Female	5 (16.67)	5 (10.64)		
**Race**, ***n*** **(%)^a^**				
Black	23 (76.67)	45 (95.74)		0.024
White	7 (23.33)	2 (4.26)		
**Smoker**, ***n*** **(%)**				
Yes	15 (50.00)	26 (55.32)	0.21	0.648
**Medication**, ***n*** **(%)**				
Atypical antipsychotic	-	32 (68.09)		
Typical antipsychotic	-	3 (6.38)		
Both atypical and typical	-	8 (17.02)		
None	-	4 (8.51)		
Age, Years, Mean (*SD*)	52.93 (10.59)	51.55 (9.05)	0.61	0.544
*PANSS, n*	-	47		
**PANSS, Mean (*****SD*****)**				
Positive symptoms	-	15.23 (4.20)		
Negative symptoms	-	16.74 (5.73)		
General symptoms	-	26.30 (5.58)		
Total	-	58.27 (11.52)		

a *Fisher's exact test used instead of Chi-sq due to small sample size*.

### Analyses of TOXO and Kynurenine Pathway Variables Between SCZ and CON Subjects

Table 2 shows the results for a comparison of TOXO status (seropositive vs. seronegative) between CON and SCZ subjects analyzed with Chi square; although percent of TOXO seropositives was higher in the SCZ group compared to the CON group, this difference was not statistically significant (Chi Square = 0.31; *p* = 0.576). TOXO serointensity was compared between CON and SCZ subjects separately for the seropositive and seronegative subjects by means of univariate ANOVAs with age, race, and sex as additional predictors. As shown in Table 2, the TOXO negatives did not differ significantly for serointensity between CON and SCZ subjects [F_(1,55)_ = 2.33, *p* = 0.132; η^2^ = 0.041]. For the TOXO seropositives with SCZ serointensity was higher than CON, although this did not reach statistical significance [F_(1,14)_ = 3.41, *p* = 0.086; η^2^ = 0.196].

**Table 2:** Kynurenines, TOXO status, and startle variables by diagnostic group.

	**Control**	**Schizophrenia**	**Chi Sq**	***p*-Value^a^**
***Toxoplasma gondii Status, n***	30	47		
Positive, *n* (%)	6 (20.0)	12 (26.67)	0.31	0.576
Negative, *n* (%)	24 (80.0)	35 (77.78)		
			*F*-value	
***Toxoplasma gondii***	30	47		
***Serointensity^b^, n*****, Mean (SD)**				
Positive, IU/mL	68.93 (22.79)	205.2 (197.1)	3.41	0.086
Negative, IU/mL	6.79 (5.49)	5.62 (2.28)	2.33	0.132
***Kynurenines^c^, n**,* **Mean (*****SD*****)**	29	45		
Tryptophan, uM	61.63 (19.26)	54.66 (11.78)	5.40	0.023
Kynurenine, uM	2.72 (0.84)	2.71 (1.03)	0.00	0.970
Kynurenic acid, nM	47.52 (40.90)	33.16 (17.02)	2.79	0.099
3-hydroxyanthranilic	29.42 (18.34)	25.85 (13.96)	0.77	0.385
acid, nM				
Anthranilic acid, nM	14.85 (18.41)	14.44 (11.51)	0.29	0.590
Kynurenine:Tryptophan Ratio	0.05 (0.03)	0.05 (0.02)	0.31	0.580
3OHAA:AA Ratio^d^^,^^e^	2.50 (1.45)	2.22 (1.20)	1.00	0.320
***Startle testing, n*****, Mean (SD)**	29	36		
Onset Latency^f^, ms	47.87 (11.22)	48.68 (12.82)	5.76	0.019
Peak Latency^f^, ms	66.55 (11.57)	70.77 (12.24)	4.34	0.041
Magnitude, μV	97.48 (74.32)	211.86 (261.81)	6.33	0.014

a *Difference between Control and Schizophrenia subjects*.

b *Covariates included: age and diagnosis (CON vs. SCZ)*.

c *Covariates included: age, sex, diagnosis (CON vs. SCZ), Toxo status (seropositive vs. seronegative)*.

d *Sample size for this metabolite was 73*.

e *3-hydroxyanthranilic acid:Anthranilic acid ratio*.

f *Covariates included: age and startle magnitude*.

Table 2 displays the means for KYN pathway variables for CON and SCZ subjects. We conducted univariate ANOVAs with each KYN pathway variable as the dependent measure, Diagnosis as the between group variable, and age, sex, and TOXO status (seropositive vs. seronegative) as covariates. Values of KYN pathway variables and results of these analyses are shown in Table 2. Plasma TRYP was significantly higher in CON than SCZ subjects [F_(1,69)_ = 5.40, *p* = 0.023; η^2^ = 0.073]. TOXO status in this model was also highly significant, with marginal means of TRYP in the TOXO+ subjects of 66.11 vs. 54.79 uM in the CON group [F_(1,69)_ = 7.74, *p* = 0.007; η^2^ = 0.101]. Other KYN pathway markers did not significantly differ between CON and SCZ subjects.

In follow up to this TOXO finding, we conducted backward linear regressions with KYN pathway variables as dependent measures and TOXO serointensity as a predictor variable. These models included Diagnosis along with age and race as additional predictors. Higher TOXO serointensity was a significant predictor of TRYP (B = 9.85, *p* = 0.001). Diagnosis was also significant in this model such that higher TRYP was associated with the CON group (B = −8.68, *p* = 0.011). Higher TOXO serointensity predicted higher values for the 3OHAA:AA ratio (B= 0.65, *p* = 0.008). Regressions predicting other KYN pathway variables were not significant for TOXO serointensity.

### Analyses of Startle Variables Between SCZ and CON Subjects

Table 2 displays the means for startle variables for CON and SCZ subjects. We conducted univariate ANCOVAs on onset and peak latency with age and log transformed startle magnitude as covariates. Race, sex, and TOXO status (seropositive vs. seronegative) as covariates were not significant so were omitted from the final models. Onset latency was slower in SCZ than CON subjects [F_(1,61)_ = 5.76; *p* = 0.019; η^2^ = 0.087]. Log transformed startle magnitude was also significant in this model [F_(1,61)_ = 35.38; *p* < 0.001; η^2^ = 0.37] and age was at a trend level of significance [F_(1,61)_ = 3.26; *p* = 0.076; η^2^ = 0.051].

In a parallel model, peak latency was also slower in SCZ than CON subjects [F_(1,61)_ = 4.34; *p* = 0.041; η^2^ = 0.066]. In this peak latency model, age was the only other significant predictor [F_(1,61)_ = 6.61; *p* = 0.013; η^2^ = 0.098].

Although startle magnitude was not a main focus of the project, we conducted a univariate ANOVA to compare CON and SCZ subjects with log transformed startle magnitude to pulse-alone trials as the dependent variable. In the final model in which only Diagnosis was retained after non-significant predictors were omitted, SCZ subjects had a larger magnitude than CON [F_(1,63)_ = 6.33; *p* = 0.014; η^2^ = 0.091]. TOXO status was not significant as a covariate in a preliminary model [F_(1,62)_ = 0.60; *p* = 0.443; η^2^ = 0.010].

We ran a similar model with log startle magnitude as the dependent variable in order to compare SCZ to CON subjects, but in this model TOXO intensity was substituted for TOXO status. Again, SCZ had higher magnitudes than CON subjects [F_(1,62)_ = 6.86; *p* = 0.011; η^2^ = 0.100]. TOXO intensity was non-significantly associated with higher magnitude [F_(1,62)_ = 1.25; *p* = 0.268; η^2^ = 0.020].

### Relationship of TOXO Serointensity to Diagnosis and Startle Variables

Because our underlying hypothesis was that TOXO serointensity could modify the relationship of latency to Diagnosis, we repeated the above models but substituted TOXO serointensity in place of TOXO status as a predictor. In the model examining onset latency as the dependent variable, Diagnosis was again significant such that latency in SCZ was slower than in CON subjects [F_(1,60)_ = 5.15; *p* = 0.027; η^2^ = 0.079]. Again, log transformed startle magnitude was a significant predictor [F_(1,60)_ = 33.87; *p* < 0.001; η^2^ = 0.36]. In the model examining peak latency as the dependent variable, Diagnosis was significant at a trend level such that latency in SCZ was slower than in CON subjects [F_(1,60)_ = 3.96; *p* = 0.051; η^2^ = 0.062]. Age was a significant predictor in this model [F_(1,60)_ = 5.76; *p* = 0.020; η^2^ = 0.088]. In these models TOXO serointensity was not a significant predictor of startle latency.

### Relationship of Kynurenines and Startle Latency

To determine whether concentrations of KYN pathway metabolites were associated with startle latency, we conducted a series of stepwise backward linear regressions with onset or peak latency to pulse-alone trials as the dependent variables and kynurenine metabolites as the predictors. In these models we also included age, sex, race, and log transformed startle magnitude as covariates. For our initial models we analyzed all subjects as a single group. The regressions on onset and peak latency were all non-significant for KYN pathway markers with the exception that lower KYNA predicted slower peak latency (B = −4.65, *p* = 0.033).

Our hypothesis was that both diagnosis and TOXO status (seropositive vs. seronegative) would affect these relationships, so we conducted these analyses with the cohort stratified first by Diagnosis (and included TOXO status as a predictor), and then by TOXO status (and included Diagnosis as a predictor). Table 3 displays significant results of these linear regressions. After stratification by Diagnosis, slower onset latency in SCZ subjects was predicted by higher TRYP (B = 0.42; *p* = 0.008), lower KYN:TRYP (B = −185.42; *p* = 0.034), and higher 3-OHAA:AA (B = 3.68; *p* = 0.007). In regressions with peak latency as the dependent variable for the SCZ group, slower peak latency was predicted by higher TRYP (B = 0.47; *p* = 0.013) and 3-OHAA:AA ratio (B = 4.35; *p* = 0.010), and by lower KYNA (B = −6.67; *p* = 0.036). In CON subjects neither onset nor peak latency was predicted by any KYN metabolites. Additional predictors that were significant are shown in Table 3.

**Table 3:** Significant models for kynurenine pathway measures and diagnosis predicting startle latency in SCZ subjects.

**KYN metabolite as predictor**	**B (SE), metabolite**	***p*-value, metabolite**	***r*^**2**^ of model**	**Other predictors in model**	**B (SE), predictor**	***p*-value, predictor**
**Onset Latency**						
TRYP	0.42 (0.15)	0.008	0.560	Magnitude	−17.63 (3.79)	<0.001
KYN:TRYP ratio	−185.42 (83.36)	0.034	0.524	Magnitude	−17.02 (4.11)	<0.001
3-OHAA:AA ratio	3.68 (1.26)	0.007	0.585	Magnitude	−21.24 (3.60)	<0.001
**Peak Latency**						
TRYP	0.47 (0.18)	0.013	0.310	Age	0.48 (0.18)	0.014
KYNA	−6.67 (3.02)	0.036	0.337	Age	0.43 (0.18)	0.025
				Race	−17.17 (7.87)	0.037
3-OHAA:AA ratio	4.35 (1.58)	0.010	0.326	Age	0.41 (0.18)	0.031

*Final models were selected using backward selection. Only metabolites with p < 0.05 are shown*.

*TRYP, tryptophan; KYN, kynurenine; 3-OHAA, 3-hydroxyanthranilic acid; AA, anthranillic acid; KYNA, kynurenic acid*.

**Figure 1:**
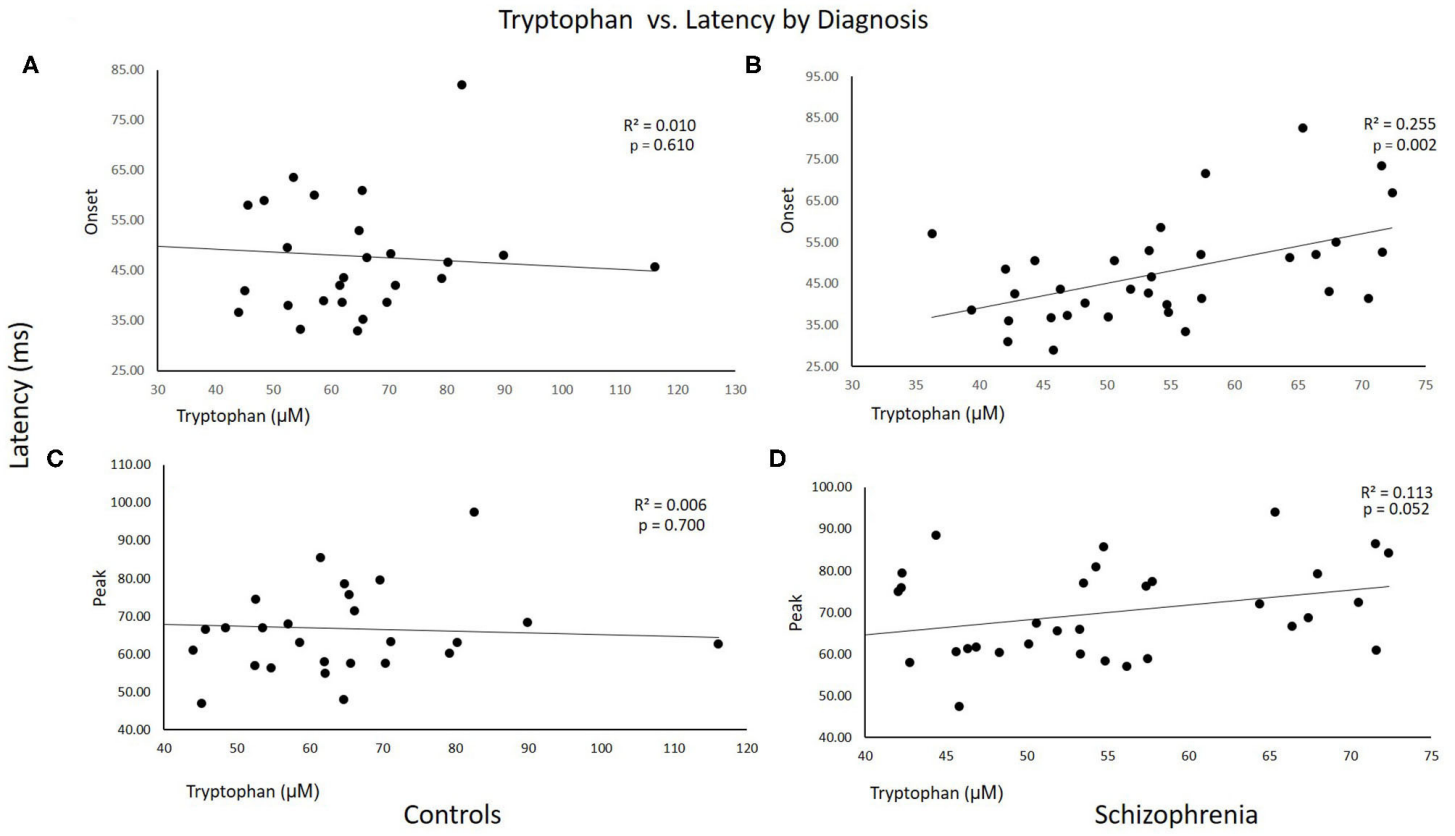
Scatterplots of tryptophan vs. startle latency for Control and Schizophrenia subjects plotted separately. *R*^2^ and *p*-values for the correlations are shown on the graphs. **(A,C)** Control subjects. **(B,D)** Schizophrenia subjects.

Figure 1 shows scatterplots of TRYP vs. startle latency for CON and SCZ subjects plotted separately. As can be seen in these graphs, the correlations were significant in SCZ but not CON subjects. *R*^2^ values indicated that TRYP accounted for 25 and 11% of the variability in onset and peak latency respectively in SCZ subjects but only 1 and 0.5% in CON subjects.

In regressions stratified by TOXO status, complementary results emerged, with statistically significant associations found only in the seropositive group. Slower onset latency was not significantly predicted by KYN metabolites. Among those in the TOXO positive group, Diagnosis was significant such that SCZ subjects had slower onset latency than CON subjects (B = 14.93; *p* = 0.018). For peak latency the regressions were more revealing (Table 4). In TOXO positive subjects, faster latency response was predicted by high KYN:TRYP ratio (B = −347.01; *p* = 0.03; and by high KYN itself (B = −8.08; *p* = 0.008) Likewise, faster latency was predicted by higher KYNA (B = −10.64; *p* = 0.003) (Table 4). Additional predictors that were significant are shown in Table 4.

**Table 4:** Significant models for kynurenine pathway measures predicting peak startle latency in TOXO seropositive subjects.

**KYN metabolite as predictor**	**B, metabolite**	***p*-value, metabolite**	***r*^**2**^ of model**	**Other predictors**	**B, predictor**	***p*-value, predictor**
**Peak Latency**						
KYN	−8.08	0.008	0.649	Race	−15.29	0.028
KYNA	−10.64	0.003	0.697	Age	0.61	0.026
KYN:TRYP ratio	−347.01	0.031	0.472	Race	−14.18	0.031

*Final models were selected using backward selection. Only metabolites with p < 0.05 are shown*.

*KYN, kynurenine; KYNA, kynurenic acid; TRYP, tryptophan*.

**Figure 2:**
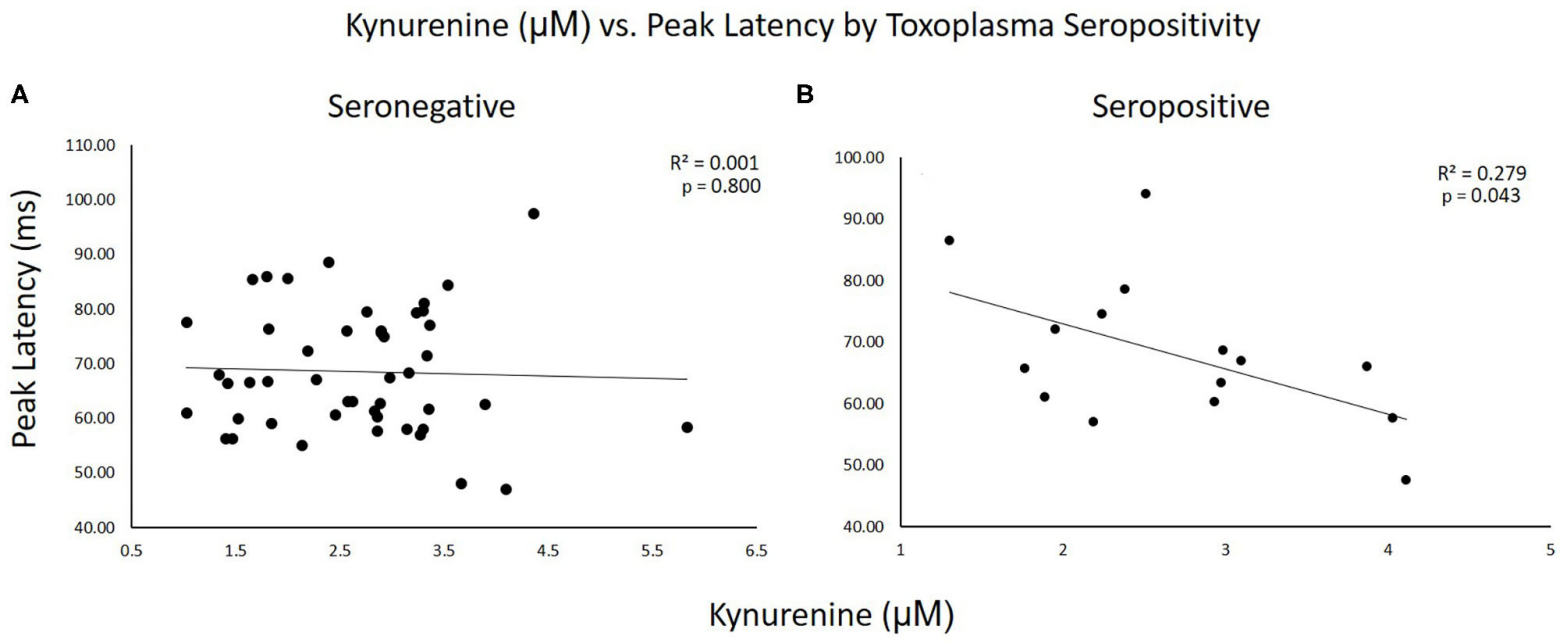
Scatterplots of kynurenine vs. startle latency for TOXO seronegative and seropositive subjects plotted separately. *R*^2^ and *p*-values for the correlations are shown on the graphs. **(A)** Seronegative subjects. **(B)** Seropositive subjects.

Figure 2 shows scatterplots of KYN vs. startle latency for TOXO seronegative and seropositive subjects plotted separately. As can be seen in these graphs, the correlations were significant in seropositive but not seronegative subjects. *R*^2^ values indicated that KYN accounted for 28% of the variability in peak latency in seropositive subjects but only 0.1% in seronegative subjects.

## Discussion

The overarching purpose of this work is to dissect the interactions of TOXO seropositivity, indicators of KYN pathway activation, and latency of acoustic startle in SCZ and CON subjects. There are several significant findings from our study and some were contrary to our original hypothesis.

Latency was slower in SCZ than CON subjects, as has been reported previously by our group and others (22–30), although in the current analysis the numerical difference in onset latency between groups was quite small. We found higher startle magnitude in SCZ than CON subjects and included this variable (log transformed) in our models comparing latency between subject groups because the magnitude difference could introduce a potential confound into the comparison of latency.

Our findings on tryptophan levels were particularly interesting. Consistent with our hypothesis, controls had higher levels of tryptophan than the SCZ group. However, the levels of tryptophan among the CON group had no relationship with startle latency, which was in contrast to the SCZ group whereby higher levels of tryptophan were associated with longer onset and peak latency. This latter finding is unexpected since tryptophan depletion leads to enhanced dopaminergic responses (46), and startle latency is prolonged under conditions of increased dopamine stimulation or activity (47–49). Increased subcortical dopamine neurotransmission has long been accepted as a mechanism underlying SCZ (50–53). We hypothesized that lower TRYP would be associated with worse (slower) startle latency, but we found the opposite in the SCZ group.

Consistent with our findings in controls, a prior study found that tryptophan depletion had no significant effect on startle latency among healthy females (54). Thus, our finding of higher tryptophan correlating longer latency in the SCZ group could be specifically-related to schizophrenia. This could be connected to medication, since the slower latency in SCZ is partially normalized by antipsychotic medication (44). Nevertheless, medication status did not have a statistically significant association with any of the kynurenine metabolites (data not shown). Given that most of patients in the SCZ group were on antipsychotic medication, this suggests that the portion of startle latency that is prolonged in SCZ and is not normalized by medication shows a positive correlation with TRYP.

Moreover, in SCZ but not CON subjects longer latency was predicted by higher values of the 3-OHAA:AA ratio and lower KYNA. A higher 3-OHAA:AA indicates greater conversion of AA to 3-OHAA among those with slower latency. In contrast to the findings of Oxenkrug et al. (55), AA was not elevated in SCZ vs. CON in our study (55). A heightened conversion of AA to 3-OHAA may be an indicator of increased neurotoxicity (56), and there is a strong correlation between plasma AA and cerebrospinal fluid (CSF) AA levels (57). The association of lower KYNA with slower latency was unexpected but highlights the complex role of this metabolite in schizophrenia pathogenesis. There is a significant correlation between plasma and CSF concentrations of KYN (58), but KYNA does not cross the blood brain barrier (59, 60).

Previous studies indicate lower blood levels of KYNA in SCZ compared to controls (61–64). We likewise found KYNA levels were 30% lower in the SCZ group vs. the CON group, although our results did not reach statistical significance. Conversely, increased KYNA levels are found in the CSF of schizophrenia patients (65–67). It is also possible that the association between slowing of latency and KYN pathway markers is an indirect one mediated by inflammation, since slowing of latency is likewise associated with elevations in inflammatory cytokines (68). Although this connection between inflammation and early steps in the activation of the KYN pathway was not borne out by our TRYP findings, we considered the immunoregulatory function of KYNA in relation to startle latency. The KYNA product of this metabolic pathway is not strictly a pro-inflammatory mediator (69, 70), and Chiappelli et al. (64) reported no significant association between common inflammatory markers and plasma KYNA (64). Additional studies will be needed to disentangle the complex relationship between inflammation, KYN pathway regulation, SCZ and acoustic startle parameters.

A related objective of this study was to examine the contribution of TOXO to the KYN pathway in acoustic startle. CON subjects had higher TRYP than SCZ, and we anticipated that TOXO infection would be associated with a lower TRYP because TOXO infection elicits an immune response that relies on induction of the cytokine IFNγ, which in turn inhibits TOXO replication by depletion of TRYP (7). However, in models controlling for CON/SCZ status, TOXO was associated with higher TRYP. We also examined TOXO serointensity, which is an indicator of the IgG response to TOXO, and is often examined in relation to SCZ (1, 71, 72). TOXO serointensity (titer) and seropositivity are obviously related, because seropositivity is simply serointensity dichotomized at a predetermined cutoff. A higher serointensity is thought to be due to a more vigorous B-cell response, perhaps due to more recent infection or greater reactivation of the organism (73). Higher TOXO serointensity predicted higher TRYP levels, and higher values for the 3OHAA:AA ratio. Finally, in TOXO positive but not TOXO negative subjects, slower latency was predicted by lower concentrations of KYN, KYNA, and a lower KYN:TRYP ratio. This underscores that high TRYP has a disadvantageous effect on startle latency whereas high KYN is associated with better (faster) startle latency.

Our interest in examining startle latency in this context stems from several factors. Startle is mediated by a simple subcortical circuit (20, 21), and is a putative index of general neural processing speed (22). The preponderance of prior studies comparing SCZ to CON subjects have reported slowing of latency in SCZ (22–25, 27, 29, 30), although there are three negative studies (31–33). Relevant to the current work, TOXO seropositive subjects have slower latency than seronegative subjects, both amongst SCZ and CON subgroups (35). Slowing of startle latency is associated with slowing of psychomotor processing (68), and psychomotor slowing in turn is well-documented to occur in SCZ (74–77). We hypothesize that slowing of neural processing speed as indicated by slow latency may disrupt the normal function of neural circuits that underlie cognitive function. Hence slow startle latency as seen in SCZ and in chronic TOXO infection may be an underlying contributor to impaired cognition in SCZ and chronic TOXO infection. Nevertheless, our findings on TRYP were overall in the opposite direction as we hypothesized.

Another possibility is that the slowing of latency we report in our SCZ and TOXO seropositive subgroups is related to hyperdopaminergia rather than to a direct causal connection with the KYN pathway. Startle latency is prolonged under conditions of increased dopamine stimulation or activity (47–49). Increased subcortical dopamine neurotransmission has long been accepted as a mechanism underlying SCZ (50–53). There is considerable evidence of KYN pathway activation in SCZ irrespective of TOXO status (61, 65–67, 78–80). The slowing of latency in TOXO positive but not TOXO negative subjects could be from simultaneous hyperdopaminergia and a complex dysregulation of the KYN pathway in the TOXO seropositive subgroup. The TOXO organism possesses two genes that code for tyrosine hydroxylase, the rate limiting enzyme for the synthesis of dopamine (81). Furthermore, this enzyme is active in the host and may be one of the factors that leads to increased dopamine synthesis, contributing to a hyperdopaminergic state (7, 15).

This work has the strength of being a novel approach to understanding the neural underpinnings of the well-replicated association of TOXO chronic infection with SCZ risk. A weakness of the study is the relatively modest sample size, particularly of the TOXO seropositive subgroup. Furthermore, the subjects were drawn chiefly from the clinical population of an urban Veterans Administration hospital and thus may not be fully representative of other populations in the United States.

In summary, this work shows that KYN pathway markers predict slowing of startle latency in SCZ subjects and in those with chronic TOXO infection, but this is not seen in CON subjects nor TOXO seronegative subjects. Follow up work in preclinical models is needed in order to further understand the meaning of KYN pathway alterations and explore potential individualized treatment targets for a subset of SCZ patients.

## Data Availability Statement

The raw data supporting the conclusions of this article will be made available by the authors, without undue reservation.

## Ethics Statement

The studies involving human participants were reviewed and approved by Emory Institutional Review Board, Atlanta Veterans Affairs Office of Research and Development. The patients/participants provided their written informed consent to participate in this study.

## Author Contributions

BP and ED designed the study and obtained funding to conduct the study. Data were collected by NM, ZG, AA, NG, BC, and MF. KYN pathway metabolites were measured by DB, PP, and VB. TOXO IgG was measured by BP, NM, and AH. The data were analyzed and interpreted by BP, NM, DG, and ED. The manuscript was written by NM, ED, and BP. All authors reviewed and approved the final manuscript.

## Conflict of Interest

ED has received research support for work unrelated to this project from Auspex Pharmaceuticals, Inc., Teva Pharmaceuticals, Inc., and is a fulltime attending psychiatrist in the Mental Health Service Line at the Atlanta Veterans Affairs Health Care System, Decatur, GA. The remaining authors declare that the research was conducted in the absence of any commercial or financial relationships that could be construed as a potential conflict of interest.
